# Managing infectious mass casualty incidents in port environments strategy development and evaluation within the ARMIHN project

**DOI:** 10.3389/fpubh.2026.1878189

**Published:** 2026-07-08

**Authors:** Nadine Sprössel, Julian Heuser, Angelina Klein, Marcus Oldenburg, Lukas Belz, Volker Harth, Lena Ehlers, Martin Dirksen-Fischer, Boris Tolg, Axel Ekkernkamp, M. Sinan Bakir

**Affiliations:** 1 Department of Orthopedics, Trauma Surgery and Rehabilitative Medicine, Department of Surgery, University Medicine Greifswald, Greifswald, Germany; 2 Central Department of Occupational Medicine and Maritime Medicine, Medical University Hamburg-Eppendorf, Hamburg, Germany; 3 Hamburg Port Health Center, Hamburg, Germany; 4 University of Applied Sciences Hamburg, Hamburg, Germany; 5 MSB Medical School Berlin, University of Health and Medicine, Berlin, Germany; 6 Helios Clinic Berlin-Buch, Center for Orthopedics and Trauma Surgery, Berlin, Germany

**Keywords:** ARMIHN project, disaster medicine, infectious disease, infectious outbreak, maritime medicine, mass casualty incident, operational strategy, port

## Abstract

**Background:**

The ARMIHN project (“Adaptives Resilienz Management im Hafen”/“Adaptive Resilience Management in the Port”) aims to develop an operational strategy for managing mass casualty incidents involving infectious diseases (MCI-ID) in port environments. The strategy considers structural, organizational, and technical measures, accounting for end-user needs and local infrastructural characteristics, with the aim of optimizing patient care and facilitating a rapid transition back to individual-level standards of care.

**Methods:**

A selective literature review identified existing national concepts, guidelines, and frameworks related to major incident management in Germany. Port infrastructural conditions were assessed through on-site inspections and stakeholder engagement. To evaluate the strategy, structured crisis management exercises were conducted, comprising three command-level tabletop exercises and one full-scale field exercise. Participant self-assessments were collected via a five-point Likert scale questionnaire before and after each exercise; pre-to-post differences were analyzed using Pearson's chi-square and Fisher's exact test where appropriate.

**Results:**

A MCI-ID operational strategy for the Port of Hamburg was developed in alignment with existing frameworks, integrating local infrastructural capacities and the expected temporal sequence of emergency resource deployment. The strategy comprises four phases: phase 1 (initial notification), Phase 2 (vessel arrival), Phase 3 (on-site rescue and medical response), and Phase 4 (transport and handover to preclinical emergency services). Exercise evaluations demonstrated statistically significant improvements across all domains, including basic MCI skills, incident management quality, confidence, and perceived team preparedness. Mean Likert scores increased from 3.33 to 4.03 in the command exercise (*p* < 0.001) and from 3.76 to 4.21 in the full-scale field exercise (*p* = 0.029), with approval rates improving by 18.2% to 63.0% across items.

**Conclusion:**

The developed strategy addresses a structural gap in existing MCI frameworks by providing a port-and maritime-specific response concept for infectious mass casualty incidents. Grounded in disaster medicine principles and refined through stakeholder engagement and repeated exercises, the framework enhances operational preparedness among involved organizations. Its phase-based architecture offers a transferable foundation adaptable to diverse MCI scenarios and pathogen types, contributing to the long-term resilience of port-based emergency management systems.

## Introduction

Ports constitute a critical component of modern infrastructure systems, fulfilling indispensable functions for the preservation of public health, safety, and the socioeconomic stability of the populations they serve (1). Their operational complexity derives from the convergence of a highly heterogeneous array of actors within a spatially confined and dynamically managed environment, encompassing commercial operators, maritime personnel, passengers, logistics providers, and regulatory agencies. This structural multiplicity of stakeholders and concurrent activities substantially amplifies both the likelihood, and the potential severity of disruptions across numerous interconnected operational domains (2–4).

The global experience with the coronavirus disease (COVID-19; SARS-CoV-2) pandemic has rendered the critical vulnerabilities of maritime environments to infectious disease outbreaks with exceptional clarity. Incidents aboard cruise vessels, in which confined spatial conditions and elevated passenger densities facilitated rapid pathogen transmission, demonstrated unequivocally that robust health security frameworks and pre-established, operationally validated response protocols are not merely advisable, but essential prerequisites for effective crisis management in port settings (5–9). In the absence of response concepts that are broadly familiar to all relevant stakeholders and systematically rehearsed under realistic conditions, significant time delays in the initiation of countermeasures become inevitable. Such delays carry the demonstrable risk of exponential pathogen propagation, avoidable clinical deterioration of affected individuals, and, ultimately, preventable mortality.

High-profile events, most notably the SARS-CoV-2 outbreak aboard the *Diamond Princess* in early 2020, have further underscored that effective containment of communicable pathogens in maritime settings is contingent upon anticipatory, evidence-informed decision-making at both the operational and strategic levels. Inadequate or delayed responses have been shown to markedly worsen outcomes not only for passengers and crew members, but also for the emergency response personnel deployed in these environments (4, 8). These cases serve as paradigmatic illustrations of the systemic consequences that arise when preparedness frameworks fail to account for the distinctive epidemiological dynamics and logistical constraints characteristic of port-based mass casualty scenarios.

The enduring relevance of this challenge is further underscored by a contemporaneous outbreak: in April 2026, a cluster of severe hantavirus disease—caused by the Andes strain (Orthohantavirus andesense) and involving three fatalities among 147 individuals on board—was identified aboard the Dutch-flagged expedition cruise vessel MV Hondius (10, 11). The outbreak necessitated multi-national coordination, medical evacuation of critically ill patients across several countries, and complex inter-governmental negotiations regarding port entry rights (11).

A mass casualty incident (MCI), as defined in the German DIN standard 13050 (12), describes a situation in which the number of injured and/or ill patients exceeds the available emergency medical resources, necessitating triage and coordinated resource management to achieve the greatest possible benefit for the affected population. It does not specify numerical MCI levels or categories. Operational classifications are regional dispatch and resource activation schemes established by individual federal states or local emergency medical service systems rather than by the DIN standard. The numerical suffix of the Hamburg MCI classification denotes the expected number of affected patients requiring coordinated emergency medical management rather than the severity of injuries or illness. The categories (MCI 5, MCI 10, MCI 25, MCI 50, MCI 100, and MCI 100+) function as predefined resource activation levels, enabling a scalable deployment of personnel and equipment according to the anticipated patient load (13). To date, mass casualty incident management concepts in Germany have, by and large, applied a unified operational framework to both traumatic injuries and infectious disease events, despite the substantially divergent therapeutic requirements, triage considerations, and critically the transmission-related consequences inherent to the latter (14–20). This conceptual conflation represents a structural deficiency with significant practical implications. Moreover, the predominant orientation of current MCI frameworks toward land-based environments fails to adequately capture the operational and infrastructural complexity of port settings, which encompass not only fixed land-side facilities but also vessels of varying size, function, and jurisdictional status. While existing singular response concepts may provide a partial structural foundation, they require systematic adaptation and targeted enhancement to adequately address the specific challenges posed by an infectious mass casualty incident, hereafter referred to as MCI-ID (MCI with infectious disease background), in the port context, incorporating the full spectrum of locally available medical and logistical resources (21).

These preparedness gaps were further substantiated through structured scientific exchange with end-users and operational practitioners conducted within the framework of the ARMIHN project (Adaptives Resilienz Management im Hafen/Adaptive Resilience Management in the Port). Across these consultations, insufficient access to actionable, context-specific information was consistently identified as a primary driver of dissatisfaction with existing response frameworks. Of equal concern was the near-complete absence of an established basis for systematic, recurrent training exercises specifically tailored to MCI scenarios within the port environment. In direct response to these identified deficiencies, the primary objective of the ARMIHN project is the development of a comprehensive operational strategy for the management of MCI-ID events in port settings. The guiding hypothesis of this work is that the implementation of such a strategy will yield both an institutionally anchored management framework for infectious MCIs and a measurably enhanced level of preparedness for hazardous-incident scenarios in the port environment.

## Methods

### Study registration, funding, and ethical approval

The ARMIHN project was funded by the Federal Ministry of Education and Research, Germany (grant no. 13N14923–13N14925). The study was prospectively registered with the German Clinical Trials Register (Deutsches Register Klinischer Studien, DRKS; registration no. DRKS00022327) and received full ethical approval from the local ethics committee of the University Medicine Greifswald, as well as from the Commission for the Ethical Evaluation of Research with Potential Dual-Use Risk (reference no. BB 051/19).

### Literature review and identification of existing concepts

Existing national and state-level concepts, guidelines, and operational frameworks pertaining to the management of major incidents were systematically identified through a selective literature search conducted across Google Scholar, PubMed, and Google Search (21). The following search terms were applied: “Mass casualty incident,” “Mass casualty of infectious diseases,” “Emergency concepts,” “Health Regulations Germany,” and “Rescue service concepts.” This search strategy enabled the identification of a range of MCI management frameworks currently in use within Germany.

The identified concepts were subsequently subjected to structured comparative analysis. Of particular relevance was the national MCI framework published by the Federal Office for Civil Protection and Disaster Assistance (Bundesamt für Bevölkerungsschutz und Katastrophenhilfe, BBK) (14), alongside the operationally distinct regional and state-level MCI frameworks applicable across the individual German federal states. In addition, documented concepts from past large-scale exercises, including those conducted by the Free and Hanseatic City of Hamburg (16), which constitutes the primary geographic focus of this case study, were incorporated to inform the development of novel operational approaches.

To complement the findings of the literature review, direct correspondence was established with designated representatives of key stakeholder organizations, including the Hamburg Port Authority and its Port Medical Service (PMS), the Hamburg Fire and Rescue Service, the Hamburg Authority for Health and Consumer Protection and economic representatives from the affected maritime sector, notably the cruise shipping industry. The synthesis of these findings informed an iterative development process for the operational strategy within the ARMIHN project framework, which was pursued through continuous multidisciplinary exchange at a series of expert workshops attended by representatives nominated by the participating stakeholder organizations.

### Infrastructural analysis and on-site inspection

To establish a comprehensive knowledge base regarding the structural and operational conditions relevant to an MCI-ID scenario, the infrastructural characteristics of the Port of Hamburg were systematically analyzed (21). An on-site inspection of the port facilities was conducted on 21 and 22 June 2019 during regular operations, with the explicit aim of gaining direct insight into prevailing workflows, spatial configurations, and operational processes at the terminal level.

The Port Medical Service was identified as the primary institutional interface between arriving vessels, in particular cruise ships, and land-based emergency response teams with respect to medical care coordination. Accordingly, PMS personnel were accompanied during routine operational duties, including vessel inspections and the formal certification of Maritime Declarations of Health upon ship arrival at the Port of Hamburg. This observational engagement provided essential contextual understanding of existing procedures and potential operational bottlenecks relevant to infectious MCI management.

### Crisis management exercises and evaluation

The operational strategy developed within the ARMIHN project was subsequently validated through a series of crisis management command exercises conducted in close collaboration with all involved stakeholder organizations. Preliminary exercises were held on 18 June 2021, 16 July 2021, and 6 August 2021. A final, comprehensive full-scale exercise was conducted over two consecutive days on 14 and 15 October 2021.

A total of 26 individuals participated in the first exercise, 14 in the second exercise, 21 in the third exercise, and 35 in the final full-scale exercise. Participants comprised all relevant stakeholders who would be involved in the management of a mass casualty incident involving infectious disease on board a vessel or within the port area, including the Hamburg Port Authority and its Port Medical Service, the Hamburg Fire and Rescue Service, the Hamburg Authority for Health and Consumer Protection, and representatives of the affected maritime sector, particularly the cruise shipping industry.

For the purpose of exercise evaluation, a thematically specialized questionnaire was developed and tailored specifically to the operational domains and strategic objectives addressed by the ARMIHN project. Although the instrument was not formally validated in a psychometric sense, its content was designed to comprehensively capture the principal areas of interest related to the evaluated strategies. The questionnaire was administered to all active participants immediately before (pre-evaluation) and immediately after (post-evaluation) each exercise, thereby enabling a structured assessment of changes in participants' knowledge, confidence, and situational awareness attributable to participation, as well as comparisons both within and across the different exercise formats.

Only participants who completed both the pre-exercise and post-exercise questionnaires were included in the analysis. Accordingly, 22 of 26 participants were included for the first exercise (response rate: 84.6%), 11 of 14 for the second exercise (78.6%), 19 of 21 for the third exercise (90.5%), and 17 of 35 for the final full-scale exercise (48.6%). Overall, 69 of the 96 exercise participants completed both questionnaires and were included in the evaluation, corresponding to an overall response rate of 71.9%.

### Questionnaire design and item development

The evaluation instrument comprised six items administered at both time points (pre- and post-evaluation), addressing participants' self-assessed competencies, confidence, and preparedness in the context of MCI and MCI-ID management. The items were formulated as first-person statements and covered the following domains (complete questionnaire in the Supplementary Data Sheet 1):

1. “I have basic skills in implementing an operational strategy in the event of a mass casualty incident (MCI/ MCI-ID).”
2. “I would describe the quality of my handling of a mass casualty incident (MCI/ MCI-ID) situation as good.”
3. “I have sufficient confidence in managing a mass casualty incident (MCI/ MCI-ID).”
4. “I have the ability to systematically assess complex crisis situations.”
5. “I have the ability to professionally evaluate complex crisis situations.”
6. “I believe that my team and/ or I are well prepared for a mass casualty incident (MCI/ MCI-ID) on a ship/ in a port.”

Although the questionnaire was not subjected to formal psychometric validation, item selection followed a structured and transparent development process. Item content was derived from a thorough review of the relevant scientific literature, with established determinants of operational preparedness and competency self-assessment incorporated in alignment with the conceptual framework and defined objectives of the ARMIHN project. This approach ensured content validity at the domain level, even in the absence of formal scale validation procedures.

### Response format, scoring and statistical analysis

Responses were recorded on a five-point Likert scale, enabling participants to evaluate each statement along a graduated continuum of agreement. Scale anchors were defined as follows: values of one (“does not apply at all”) and two (“mostly does not apply”) reflected disagreement; a value of three indicated a neutral stance; and values of four (“mostly applies”) and five (“fully applies”) expressed agreement with the respective statement.

For the purpose of statistical analysis, continuous Likert responses were dichotomised into binary outcome categories. The threshold was set at ≥4 (agreement) vs. ≤ 2 (non-agreement). This dichotomisation strategy was applied consistently across all items to facilitate uniform comparative analysis between pre- and post-evaluation responses.

All statistical analyses were performed using IBM SPSS Statistics for Windows (Version 26.0; IBM Corp., Armonk, NY, USA). Associations between categorical variables were assessed primarily using Pearson's chi-square test. In instances where expected cell frequencies fell below *n* = 5, Fisher's exact test was applied as the appropriate alternative to ensure analytical validity. A two-sided significance level of *p* < 0.05 was adopted as the threshold for statistical significance throughout all analyses. To facilitate interpretation of the practical relevance of observed pre–post changes, effect sizes were additionally calculated using Cohen's *d* for paired measurements based on the original five-point Likert-scale responses.

## Results

### Current state of operational preparedness in port settings

At the outset of the ARMIHN project, no established operational concepts were found to exist for the management of an MCI and specifically an MCI-ID in the port environment, neither with respect to the rescue and medical care of acutely ill or infectious individuals, nor in relation to affected cruise vessels. Structured exchange with end-users and operational stakeholders consistently identified the absence of inter-organizational standard operating procedures (SOPs) as a recurring and primary source of dissatisfaction among the parties involved. The lack of a comprehensive overarching concept shared across all relevant stakeholders not only represented a critical gap in operational readiness, but also precluded the establishment of a systematic basis for recurrent, scenario-specific training exercises addressing MCI events in the port context.

To date, no documented operational experience with an infectious MCI had been recorded within the Port of Hamburg. While individual stakeholder organizations may have maintained internal response frameworks applicable to their own operational domain, no formalized conceptual exchange or inter-organizational coordination mechanism had been established among the relevant actors. These findings collectively underscored the urgent need for a unified, port-specific operational strategy—a gap that the ARMIHN project was designed to address.

### Development of a generally applicable operational strategy

**Figure 1:**
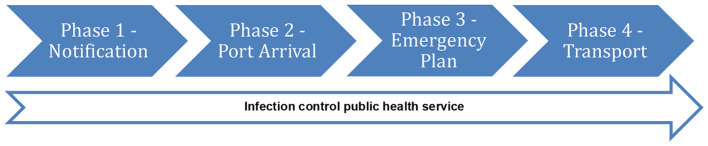
Strategy phases.

In response to the identified preparedness deficits, the ARMIHN project developed a comprehensive operational strategy for the management of MCI-ID events in the port setting. The strategy is structured into four sequential phases, derived from the specific infrastructural conditions of the port environment and the anticipated chronological arrival sequence of emergency and support personnel (Figure 1). Its conceptual foundation draws upon established frameworks and evidence-based recommendations for general MCI management (22–30), integrating individually validated concepts into a unified, coherent structure. A key advantage of this approach is its broad operational applicability: the recommended procedural steps are, in principle, transferable across diverse MCI scenarios, including infectious disease outbreaks occurring aboard ships or within the wider harbor area. The overall rescue and response process is characterized by a dual-track assessment structure, comprising both a medical sighting component and a public health authority sighting component. The phased architecture of the strategy enables each constituent phase to be examined, planned, and optimized independently, while preserving the coherence and continuity of the overall response process (30–32).

The four phases are defined as follows. **Phase 1** encompasses all preparatory measures required prior to the arrival of the affected vessel at the terminal or its designated outer area, ensuring that all necessary resources, personnel, and command structures are mobilized in advance. **Phase 2** is initiated upon the arrival of the affected ship and is characterized by the establishment of the command structure and the formation of sub-deployment sections. **Phase 3** follows directly from Phase 2 and involves the deployment of a dedicated boarding team, comprising at minimum a physician and a paramedic, or personnel with equivalent qualifications, to assess and manage patients directly on board the vessel. **Phase 4** addresses the coordinated transport of patients and identified contact persons from the ship to appropriate land-based care facilities. Throughout all four phases, and operating in parallel to the sequential response activities, the public health service maintains continuous involvement to ensure robust contact tracing and epidemiological surveillance.

#### *Phase 1*—*Notification of sick persons on board*

The first phase is initiated by the ship's master, who is obligated to submit a formal notification, typically in the form of a Maritime Declaration of Health, to the competent public health authority. In Germany, this process is conducted electronically via the National Single Window platform (33, 34). Where available, the declaration is countersigned by the ship's physician. Submission is required no later than 24 h prior to entry into the port area and must include comprehensive data on all persons on board, a complete record of ports of call within the preceding 30 days, and all documented information pertaining to illness or death occurring during the voyage. In the event of any change in health status or passenger condition, the declaration must be updated continuously to reflect the most current information available.

The 24-h notification window represents the earliest opportunity and the critical starting point for initiating preparatory measures in anticipation of a potential mass influx of sick or infectious persons into the port area (34). Upon receipt of the notification, the information must be disseminated without delay to all relevant stakeholders, including port controllers, shipping companies, terminal operators, and other operational personnel. It is important to note that, for vessels still at sea at the time of notification, primary responsibility for medical and nautical coordination rests with the Central Command for Maritime Emergencies (Havariekommando), the German authority responsible for organizing and regulating maritime security in the North Sea and Baltic Sea regions (35). Both nautical and medical expertise relevant to the vessel's situation are coordinated through this organization until jurisdiction transitions to port-based authorities upon arrival.

**Figure 2:**
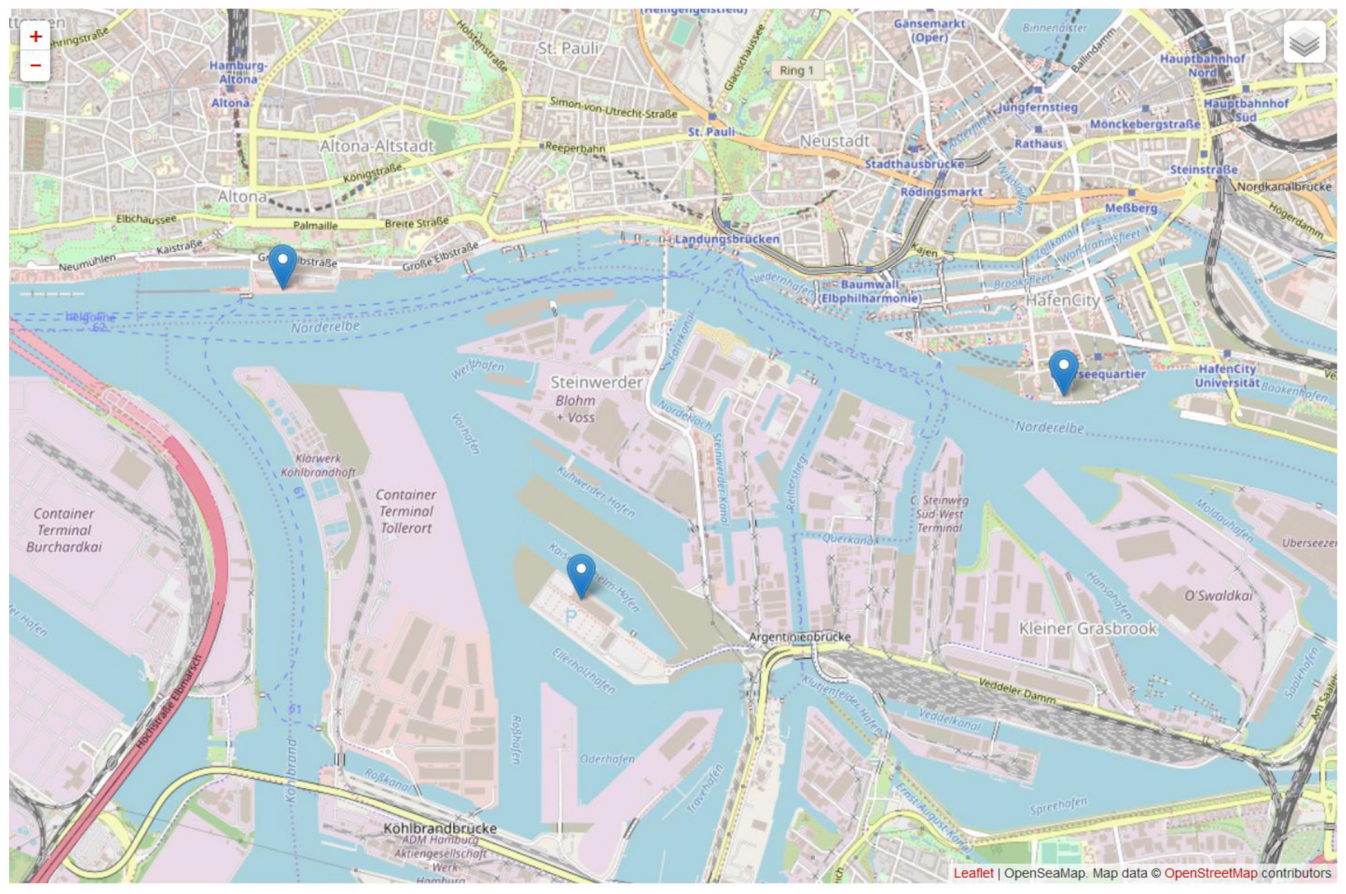
Cruise terminals of the port of Hamburg.

In accordance with the developed operational strategy, the relevant rescue services are to be alerted during Phase 1, with explicit communication that a large number of sick or potentially infectious individuals is expected. This early notification is intended to ensure that adequate background capacities are placed on standby and that alert levels are adjusted accordingly. Concurrently, the designated terminal area is to be prepared for the arrival of the affected vessel. In the case of the Port of Hamburg, three terminal locations are available for this purpose (see Figure 2). Upon completion of information dissemination and the organizational preparations characteristic of Phase 1, all involved actors are positioned to transition directly to Phase 2, with key personnel already deployed on site.

#### *Phase 2*—*Arrival at port*

Phase 2 is initiated upon the docking of the affected vessel at the designated terminal. Once the operational sections have been established in accordance with the MCI concept of the Hamburg Fire and Rescue Service, the emergency response framework becomes fully operational (16). In the context of an MCI-ID, overall command of the operation is vested in the Leading Emergency Physician (LEP). Given the specific hazardous nature of an infectious scenario, the active involvement of the Port Medical Service is considered essential to ensure informed and appropriate decision-making. The PMS fulfills a dual function in this context: it is responsible for assessing the risk to public health and for initiating all necessary measures for outbreak investigation and control (34), while simultaneously serving as an expert advisor and operational mediator, drawing upon its specialist knowledge of the infrastructural particularities and regulatory framework of the Port of Hamburg.

Depending on the nature and severity of the infectious situation, the deployment concept for Phase 2 provides for the terminal building to be designated as the primary patient treatment area—a measure particularly indicated in cases involving highly infectious, non-self-limiting diseases. Where operationally necessary, a patient staging area may additionally be established on board the vessel itself. It must be noted, however, that the structural constraints and spatial limitations inherent to cruise ships preclude a direct one-to-one transfer of land-based MCI triage and treatment configurations; context-specific adaptations are therefore required. Within the on-board environment, the ship's infirmary and hospital facilities are designated as the appropriate setting for more severely ill patients, while individuals presenting with mild symptoms are to be managed under cabin isolation in accordance with the applicable isolation regulations governing cruise vessels.

Systematic data collection by response personnel on board constitutes a critical component of Phase 2 operations. All reported cases are to be clinically assessed by physicians and qualified staff from the PMS and/or the deployed rescue services, using a standardized sighting algorithm, preferably administered via electronic devices such as tablets to facilitate real-time data capture and transfer. Each patient is assigned a triage category, which is communicated by means of a tag card or identification bracelet. The resulting data are to be relayed to the relevant command levels either through direct exchange or via radio communication. In this regard, it should be noted that wireless network connectivity within ship structures is frequently insufficient to support reliable external data transmission, making radio intercom the preferred communication modality in this setting.

The primary treatment area is established within the terminal building, where connection to mains infrastructure substantially reduces the logistical burden on operational sub-units, allowing deployed emergency services to concentrate their capacities on the core clinical task of patient rescue and care.

A specific operational contingency must be addressed for scenarios in which the infectious situation becomes apparent only late in the docking process and the number of affected patients has escalated to MCI level within a compressed timeframe due to the highly contagious nature of the pathogen involved. Under these circumstances, the sequential distinction between Phase 1 and Phase 2 is no longer operationally viable (Figure 1). In such cases, the preparatory phase is necessarily omitted, and both phases must be executed concurrently. This requires the simultaneous implementation of targeted information dissemination, immediate deployment of preparatory measures, requisition of additional emergency service resources, and the marking and establishment of designated treatment areas and access routes, all conducted in parallel rather than in sequence.

####  *Phase 3—Emergency plan*

Phase 3 is characterized by the activation and implementation of pre-established concepts and operational plans for the management of infectious patients. The emergency plan is structured around two fundamental components: an organizational-tactical level, which addresses the spatial configuration of the response area and the directed flow of patients toward the designated treatment zones; and a medical level, which encompasses clinical screening, initial treatment, and the subsequent determination of transport disposition or inpatient continuation of care. Together, these components converge in a disease-specific prioritization algorithm for infectious diseases.

#### Medical sighting and triage

To maximize the transferability of the screening algorithm and to ensure its applicability across a broad range of infectious scenarios, the triage framework was deliberately developed without reference to any specific exemplary disease. This approach reflects the operational reality that definitive pathogen identification is frequently unavailable in the preclinical setting. Consequently, the initial assessment must proceed from general clinical syndromes, such as respiratory illness, toward more specific diagnoses, including influenza, rhinovirus infection, COVID-19, or other etiologies. The algorithm was therefore designed around clinical syndromes rather than pathogen-specific diagnoses.

**Figure 3:**
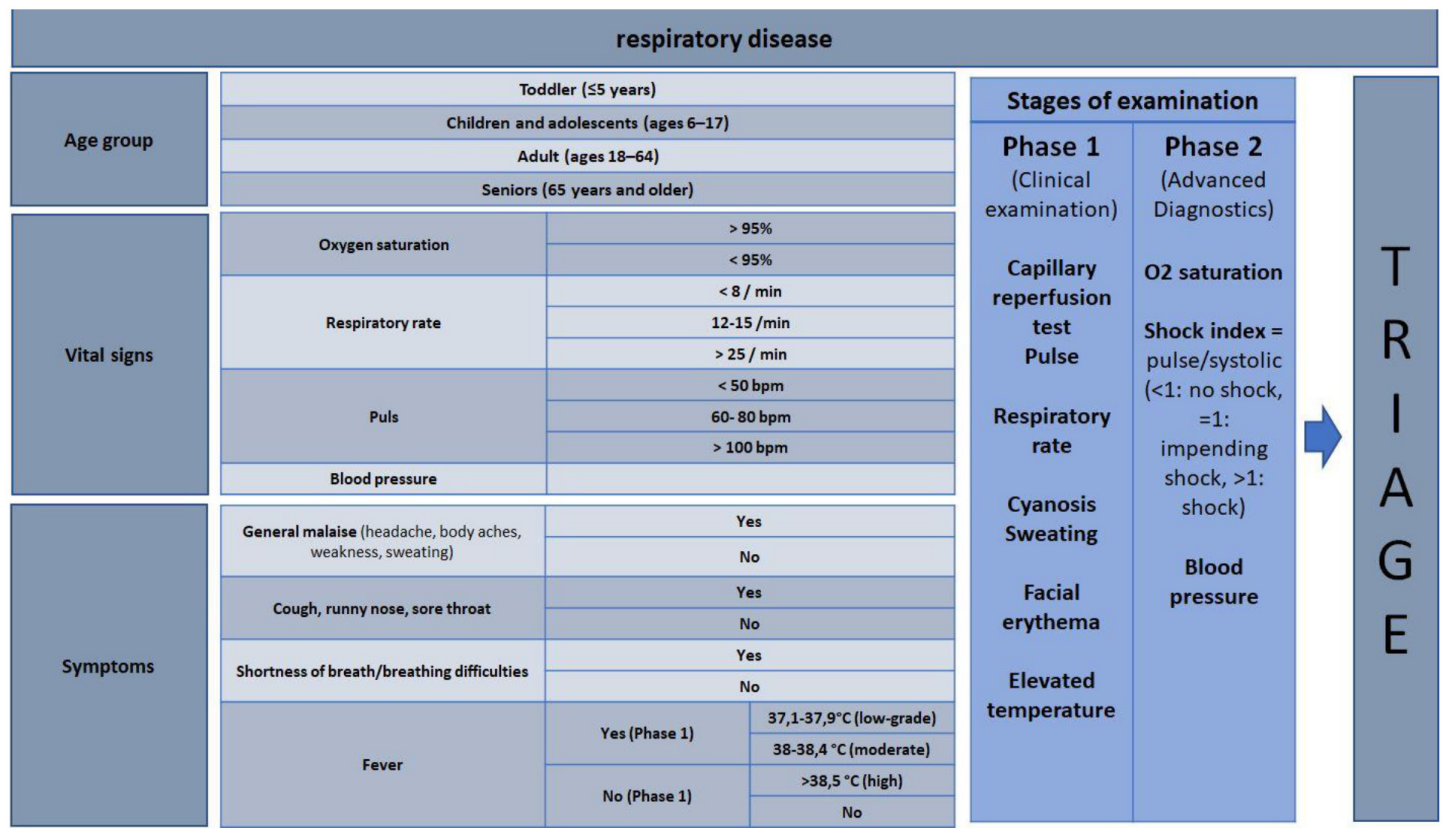
Triage algorithm developed in this study, illustrated by the example of a respiratory tract infection.

**Figure 4:**
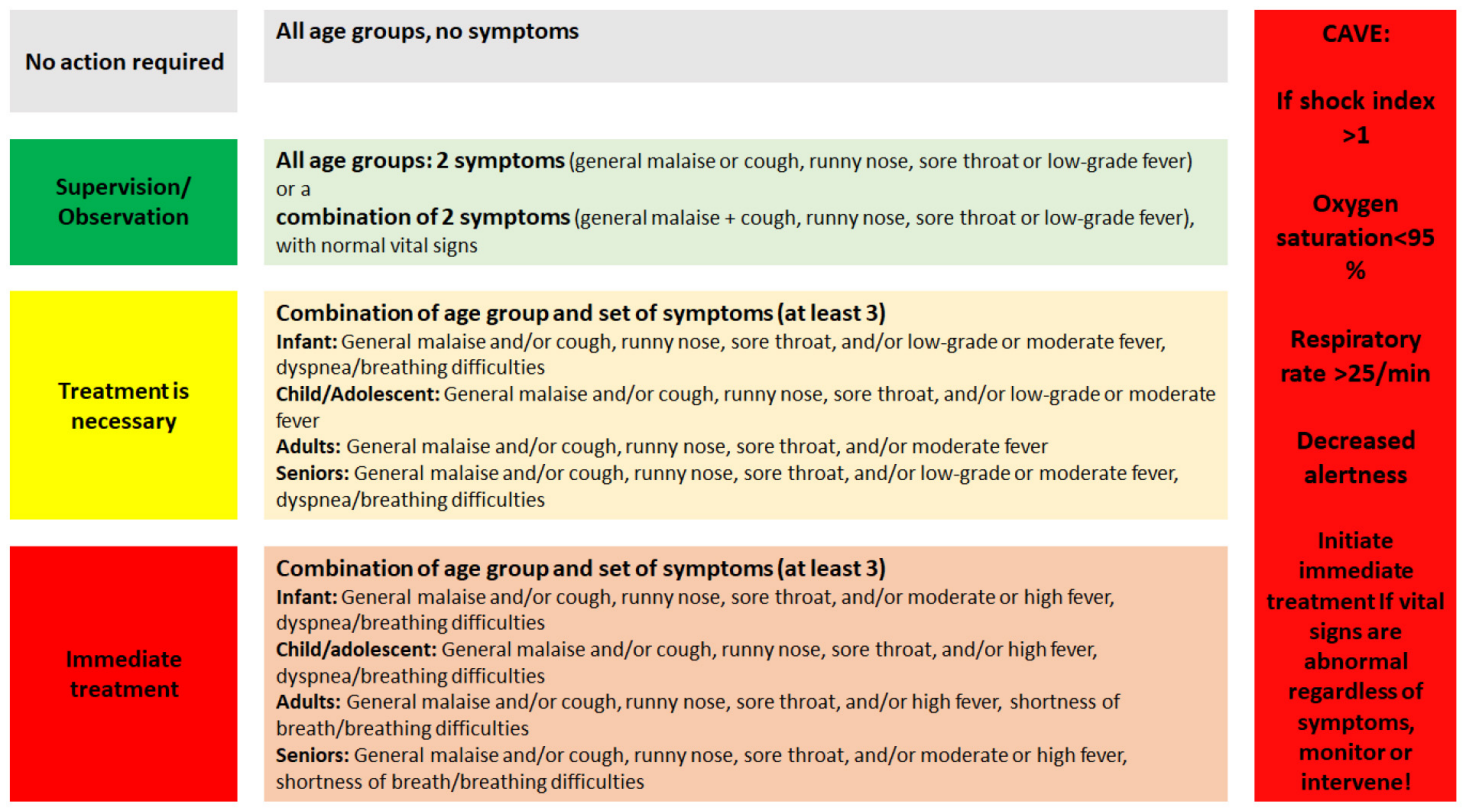
Disease-specific triage categories developed in this study, illustrated by the example of a respiratory tract infection.

With the objective of enabling rapid and straightforward screening of infectious cases aboard ships and within the port environment, a simplified prioritization scheme was developed, drawing upon the sighting categories established by the BBK (PRIOR algorithm) and the mSTaRT triage system (36, 37). The triage process is illustrated using the example of a respiratory disease (Figure 3). Figure 3 depicts the conceptual structure of the triage framework and the parameters incorporated into the assessment process, whereas the resulting prioritization criteria and category assignments are presented in Figure 4.

The triage framework is based on three principal domains: age group, vital signs, and disease-specific symptoms. Four age categories were defined ( ≤ 5 years, 6–17 years, 18–64 years, and ≥65 years) to account for differences in vulnerability and physiological response patterns among patient populations. This age stratification was considered particularly important because age has been identified as a relevant factor influencing susceptibility and disease severity in many infectious disease scenarios. Vital sign assessment comprises respiratory rate, pulse rate, blood pressure, and oxygen saturation. Threshold values for these parameters were established by expert consensus and informed by the validated Mainz Emergency Evaluation Score (MEES) (38). While age and physiological parameters are applicable across all infectious disease scenarios, the symptom domain is disease-specific, reflects the characteristic clinical presentation of the causative pathogen, and can be adapted to different infectious disease scenarios while maintaining the same general assessment structure.

For respiratory disease scenarios, symptom assessment includes general malaise, such as headache, myalgia, weakness, and sweating, as well as cough, rhinorrhoea, sore throat, dyspnoea, and fever. Symptoms are recorded in a binary manner as either present or absent.

The triage algorithm is structured as a two-stage assessment process referred to internally as Phase 1 and Phase 2. These stages are distinct from, but integrated within, the broader four-phase operational framework. Phase 1 corresponds to a preliminary triage stage and is conducted by first responder units upon initial boarding of the vessel. During this stage, patients are assessed through clinical examination alone without advanced diagnostic equipment. Evaluation includes pulse rate, respiratory rate, observation of cyanosis and facial erythema, capillary refill assessment, palpation for elevated body temperature, and simultaneous symptom screening. The primary objective of this stage is the rapid identification of potentially life-threatening conditions and the early prioritization of patients requiring immediate medical attention.

Phase 2 corresponds to the formal triage stage and is conducted after transfer to the preclinical rescue environment ashore. At this stage, the initial assessment is validated through additional physiological measurements, including oxygen saturation and blood pressure monitoring. Based on these findings, the initial triage categorization may be confirmed or revised. Operationally, Phase 1 is intended to be performed on board using electronic tablet-based documentation, whereas Phase 2 focuses particularly on the reassessment of patients initially assigned to the red and yellow categories.

The prioritization process follows an escalating logic in which the accumulation of symptoms, age-related vulnerability factors, and physiological abnormalities progressively increases the assigned triage priority. The combination of age group, physiological parameters, and symptom constellation ultimately determines the final triage category. Patients are categorized into three visual priority classes, red, yellow, and green, reflecting their current clinical status (Figure 4).

Patients classified as red require immediate life-saving interventions and rapid transfer to an appropriate hospital facility. Patients assigned to the yellow category require medical assessment and treatment but do not present with immediately life-threatening conditions. Green-category patients do not require urgent medical intervention and may be managed through observation, outpatient follow-up, or precautionary monitoring, depending on the operational situation.

In addition, Figure 4 identifies critical warning signs (“CAVE” criteria) that necessitate immediate medical intervention regardless of the underlying infectious disease and may also apply to non-infectious medical emergencies. The figure therefore translates the assessment parameters collected during the two-stage triage process into operational treatment priorities and patient management pathways.

No dedicated blue (expectant) category was incorporated into the ARMIHN triage framework. This decision reflects the specific characteristics of infectious mass casualty incidents, in which the identification of patients with no realistic prospect of survival is often considerably more uncertain than in trauma-related incidents. Clinical deterioration in infectious diseases may occur dynamically and, in some cases, remains reversible with timely supportive treatment. Consequently, patients presenting with life-threatening conditions are assigned to the red category and prioritized for immediate medical assessment and intervention. This approach was further supported by stakeholder consultations conducted during the development and evaluation phases of the project, which highlighted both operational and ethical concerns regarding the use of a separate expectant category in the confined environment of a vessel or port terminal.

#### Contact tracing within the framework of infection control

In the context of an MCI-ID aboard a vessel, the systematic identification of contact persons within the framework of infection control constitutes a task of the highest operational priority, and is accordingly integrated into Phase 3 of the emergency plan in parallel with medical triage. The infection-epidemiological assessment and the initiation of corresponding public health measures are the responsibility of the competent authority. In the Free and Hanseatic City of Hamburg, this function is fulfilled by the Port Medical Service, operating as a specialized sub-unit of the Hamburg Port Health Center.

**Figure 5:**
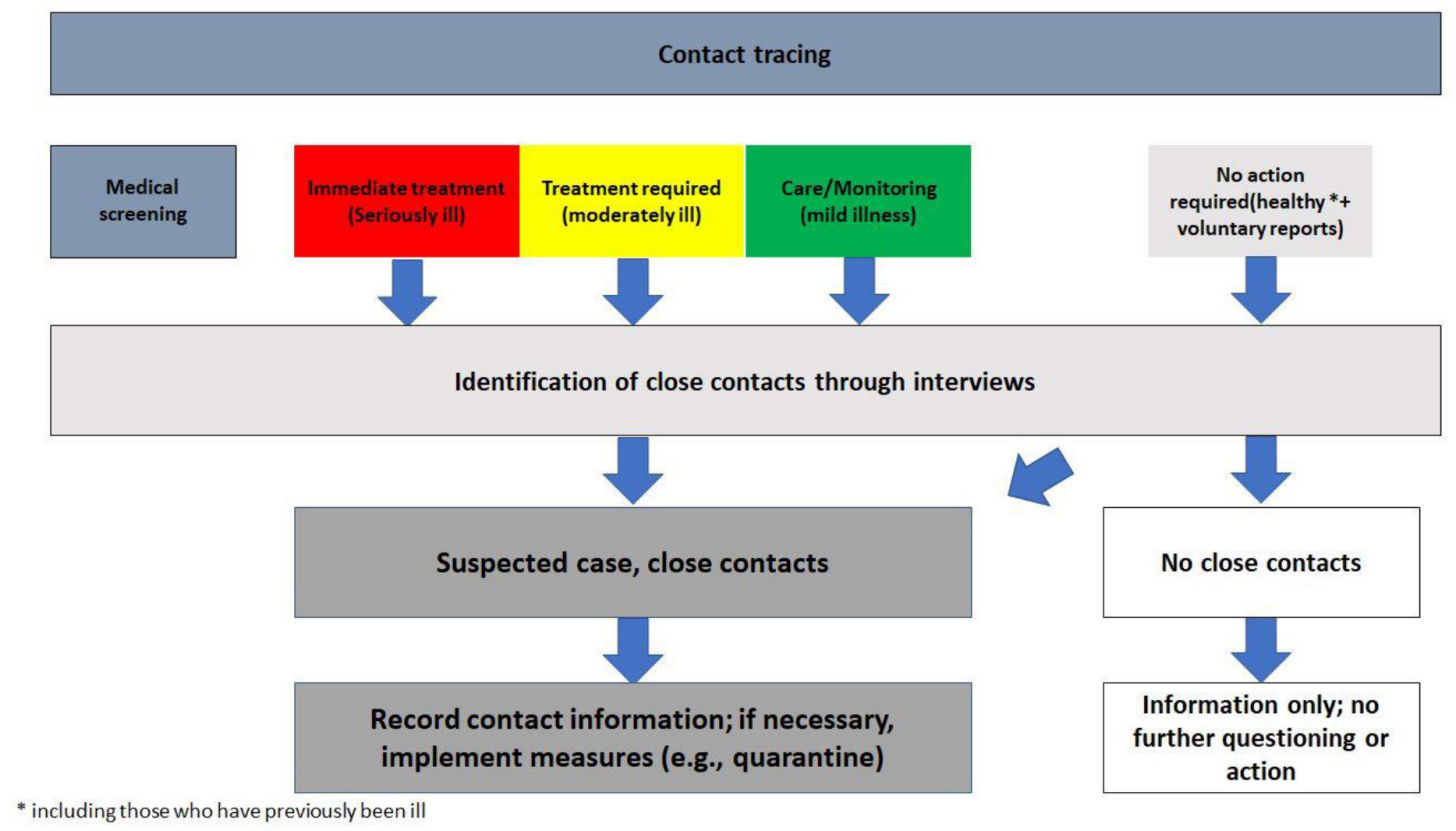
Contact tracing framework developed in this study for the identification and management of cases and contacts during a MCI-ID.

In accordance with the German Infection Protection Act (Infektionsschutzgesetz, IfSG), the statutory tasks of the PMS encompass the conduct of outbreak investigations, the systematic collection of information regarding the source of infection and transmission routes, and the identification of potential contact persons (3, 39, 40). On this basis, the PMS is empowered to order targeted public health measures, including vaccination, post-exposure prophylaxis, isolation and quarantine, the mandatory use of appropriate personal protective equipment, and disinfection and decontamination procedures. Furthermore, the PMS fulfills a critical interface function, mediating between the ship-based response and the shore-based rescue forces. While the principal volume of infection control activity follows the completion of medical triage, the situational assessment and the initiation of public health protective measures are logically commenced in parallel from Phase 1 onwards (Figure 5). Early identification of suspected cases and contact persons is of particular importance for limiting the spread of highly contagious and/or highly pathogenic infectious diseases, and should therefore be initiated on board wherever operationally feasible, and no later than at the point of passenger disembarkation.

For the practical implementation of infection control and to ensure adequate protection against onward transmission, a supplementary categorization system, distinct from the medical triage color scheme, was developed for contact tracing purposes. Persons are classified as either white (healthy, not identified as a contact person) or dark grey (suspected case or identified contact person). Individuals disembarking in Hamburg who are neither clinically ill nor identified as suspected cases or contact persons are assigned to the white category and are permitted to leave the premises without further restriction (Figure 5). To expedite the disembarkation process, relevant declaration cards and information materials are to be distributed and collected on board prior to disembarkation. Persons who are currently ill, or who were ill during the voyage but subsequently recovered, are automatically assigned to the contact/suspected case category. Voluntary self-disclosure of contact with ill persons, for example, due to shared use of communal spaces, likewise results in assignment to this category.

#### Spatial organization and patient flow

Following triage, the management of affected persons is structured according to both spatial and sequential principles. Designated areas within the port facility are assigned distinct functional roles, directing patient flow in accordance with clinical priority classification. Transfer from the vessel proceeds in a defined order, ensuring that critically ill patients are addressed first, followed sequentially by those requiring active treatment and, finally, by individuals presenting with no or only minor symptoms. Decontamination measures are applied at defined transition points between operational zones.

The allocation of patients to appropriate medical facilities is coordinated jointly by the rescue coordination center and the incident command structure. Health registration and, where indicated, contact tracing are conducted by the public health authority within designated areas of the terminal building. Depending on individual categorization, persons are either subject to further clinical or epidemiological assessment, or, in the absence of an imposed quarantine measure, permitted to leave the terminal without additional intervention.

In view of the critical infrastructure status of the Port of Hamburg and the potential dual-use risk associated with detailed operational information, specific spatial configurations and tactical deployment details are not disclosed in this publication.

####  *Phase 4—**Patient transport***

Phase 4 is defined by the coordinated implementation and operational securing of patient transport from the port facility to receiving medical institutions. Transport is executed in direct coordination among the participating command staffs, the rescue coordination center, and the designated receiving hospitals, and proceeds in descending order of clinical priority, commencing with patients assigned to the red triage category.

Pre-clinical transport is exclusively provided by the deployed emergency medical services. To ensure efficient and unimpeded access, ambulances are granted direct entry to a dedicated side entrance of the Port Medical Service duty station, in accordance with the pre-established emergency plan. The maintenance of an orderly and clearly defined traffic flow structure for both arriving and departing ambulances is considered an essential operational requirement, as uncoordinated vehicle movements risk mutual obstruction and may introduce avoidable delays into the overall response process.

The selection of receiving hospitals was determined on the basis of three principal criteria: geographical proximity to the port facility, the availability of appropriate specialist departments capable of managing infectious disease presentations of varying severity, and confirmed capacity for emergency treatment (21). This pre-selection ensures that transport destinations are established prior to the onset of the incident, minimizing decision latency during active operations and facilitating the seamless continuation of care beyond the pre-clinical phase.

### Crisis management exercises within the ARMIHN project

Within the framework of the ARMIHN project, a total of four crisis management exercises were conducted and subsequently subjected to structured evaluation. These comprised three command-level tabletop exercises and one comprehensive full-scale field exercise carried out at the port area of Hamburg, all of which took place between June and October 2021. The first and third command exercises were primarily designed to assess inter- and intra-organizational communication processes among the participating stakeholder organizations. In contrast, the second command exercise and the subsequent full-scale field exercise were specifically directed at evaluating the practicability and effectiveness of the operationally implemented strategies developed within the ARMIHN project framework. Because the primary objective of the present manuscript is to assess the operational strategy and its evaluation, only data from the second exercise and the final full-scale exercise were included in the analyses presented here.

#### Second command exercise

**Figure 6:**
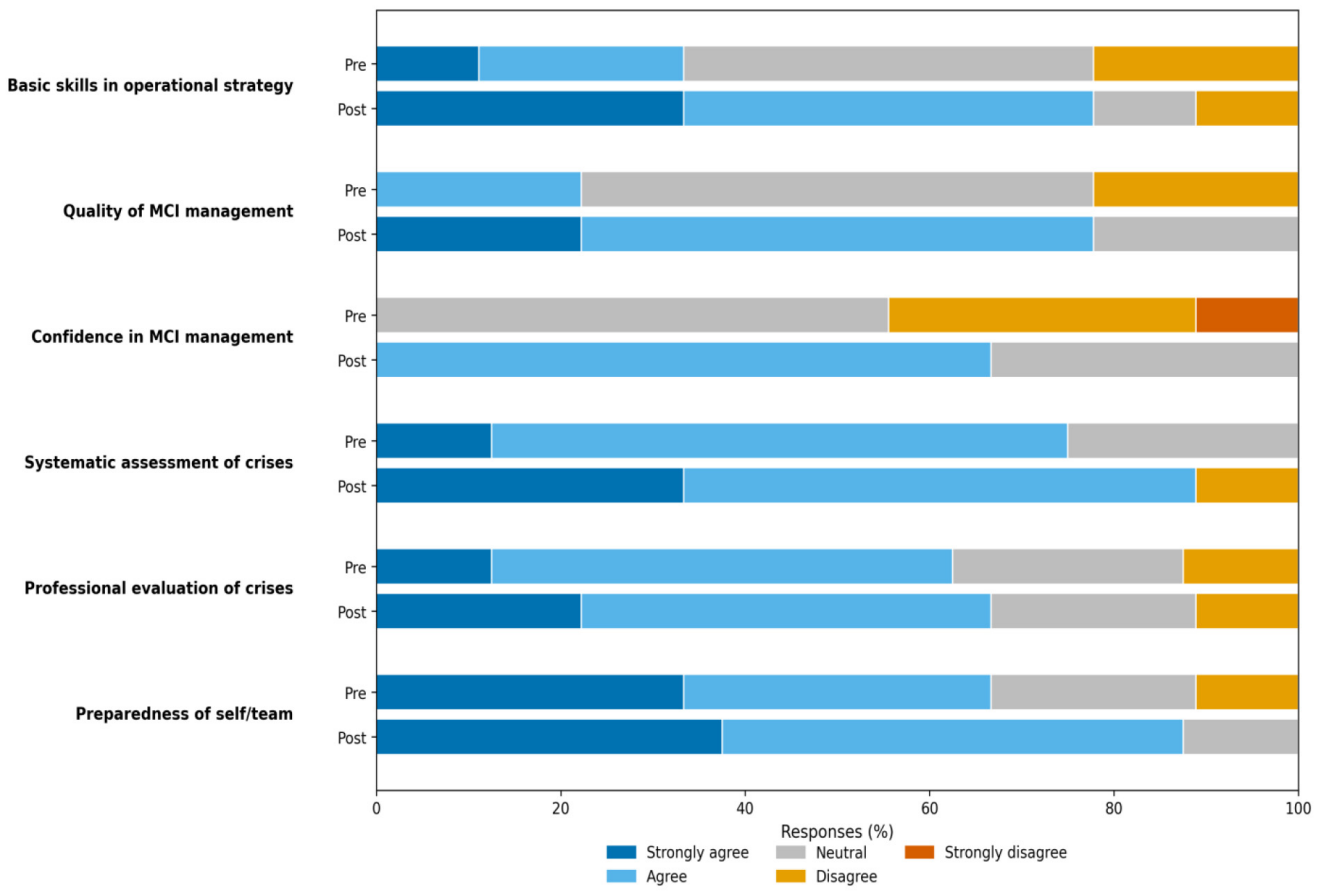
Command exercise pre- and post-evaluation.

A total of 11 participants completed both the pre- and post-evaluation questionnaires in full during the second command exercise; incomplete responses were excluded from the analysis. Pre-evaluation responses yielded a mean overall score of 3.33 (corresponding to a neutral stance on the applied five-point Likert scale), which increased significantly to a mean of 4.03 (indicating general agreement) in the post-evaluation assessment (*p* < 0.001; Figure 6). This statistically significant improvement reflects a meaningful shift in participants' self-assessed competencies and preparedness attributable to participation in the structured exercise.

### Full-scale field exercise

**Figure 7:**
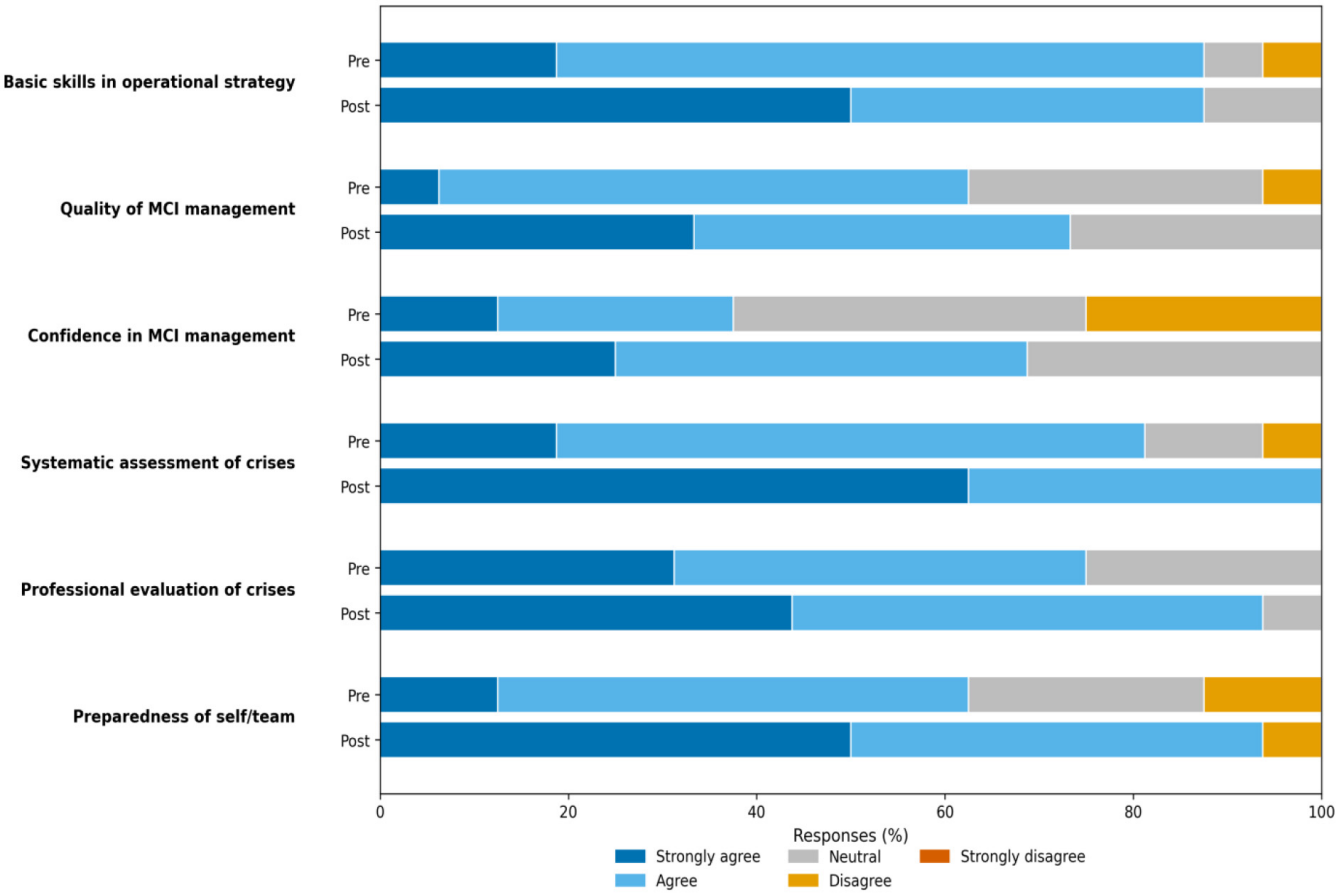
Full-scale field exercise pre- and post-evaluation.

During the full-scale field exercise conducted in hybrid format, 17 participants provided complete responses to both the pre- and post-evaluation questionnaires. Participants who completed only one of the two assessment time points were excluded from the paired analysis, yielding a total of 204 valid item-level responses (17 participants × 6 items × 2 time points). The overall mean score increased significantly from 3.76 at pre-evaluation to 4.21 at post-evaluation (*p* = 0.029; Figure 7), indicating a statistically significant and clinically meaningful improvement across the evaluated domains following exercise participation.

To facilitate interpretation of the practical relevance of the observed changes, effect sizes were additionally calculated using Cohen's *d* for paired measurements based on the original five-point Likert-scale responses. The second command exercise demonstrated a moderate effect size (*d* = 0.64), indicating a meaningful improvement in participants' self-assessed competencies and preparedness following exercise participation. Similarly, the full-scale field exercise yielded a moderate effect size (*d* = 0.56), supporting the observed increase in perceived knowledge, confidence, and preparedness.

Across all six questionnaire items, general levels of approval increased between the pre- and post-evaluation time points, while rates of rejection either decreased or were entirely eliminated.

The most pronounced gains were observed for subjective confidence in MCI management (Item 3), for which no agreement had been recorded at pre-evaluation, compared to 63.0% at post-evaluation, and for self-assessed quality of MCI management performance (Item 2), where agreement rose by 54.5% and pre-evaluation rejection was entirely eliminated. Basic skills in implementing an operational deployment strategy (Item 1) likewise showed a substantial increase in agreement of 36.4%, accompanied by a halving of the rejection rate from 18.2 to 9.1%. For the ability to systematically assess and professionally evaluate complex crisis situations (Items 4 and 5), agreement increased by 18.2% in each case, building upon already high pre-evaluation baselines of 72.7%. Similarly, perceived preparedness of self and/or team for a port- or vessel-based MCI (Item 6) improved by 18.2%, with rejection absent entirely at post-evaluation compared to 9.1% pre-evaluation.

Taken together, the evaluation data from both the second command exercise and the full-scale field exercise demonstrate consistent and statistically significant improvements in participants' self-assessed competencies, confidence, and perceived preparedness following structured exposure to the ARMIHN operational strategy. These findings provide preliminary evidence in support of the validity and practical effectiveness of the developed framework for MCI and MCI-ID management in the maritime and port-related emergency context.

## Discussion

### Development and contextual rationale of the operational strategy

The development of an operational strategy for managing a mass casualty incident in the port environment must be regarded as an intrinsically complex undertaking, situated at the intersection of disaster medicine, maritime logistics, and critical infrastructure governance. The multitude of involved actors, the existence of pre-established guidelines and regulatory frameworks, and the overarching context of critical infrastructure protection collectively generate a highly heterogeneous set of operational, legal, and organizational requirements. In such multi-layered environments, the translation of conceptual disaster response frameworks into operationally feasible procedures represents a persistent challenge.

Any newly developed strategy must therefore be rigorously evaluated with respect to its practical effectiveness, functional adequacy, and real-world applicability, with particular emphasis on end-user acceptance. Operational concepts that fail to achieve practitioner buy-in or do not demonstrably improve upon established workflows are unlikely to be sustainably implemented within complex, multi-agency emergency systems. Accordingly, existing regional MCI concepts, including those of the Hamburg professional fire and rescue service, as well as national guidance issued by the Federal Office for Civil Protection and Disaster Assistance were systematically reviewed and integrated into the strategy development process (14, 16–20, 41, 42).

The elaboration of evidence-informed strategies for the management of infectious MCIs, developed in close alignment with the operational needs and expectations of end-users, contributes substantially to preparedness across all involved stakeholders. Within disaster medicine frameworks, preparedness is a key determinant of response effectiveness and directly correlates with systemic resilience. Within this context, the Port of Hamburg represents a strategically critical interface between maritime and land-based emergency response systems, while simultaneously integrating heterogeneous organizational actors across civil protection, public health, and emergency medical services.

### Exercise evaluation, evidence of system improvement, limitations of the evaluation

The evaluation data consistently demonstrated meaningful differences between pre- and post-exercise assessments across both analyzed exercise scenarios. In both cases, mean Likert scores increased from the neutral range (level 3) to the agreement range (level 4), reflecting a measurable improvement in participants' self-assessed competence and situational awareness in MCI management. From a disaster medicine perspective, such shifts in perceived readiness are considered relevant indicators of training effectiveness, particularly in inter-organizational simulation environments.

A notable longitudinal effect was observed in baseline pre-evaluation scores across the exercise series. While the first command exercise recorded responses as low as level 1 (strongly disagree), the subsequent full-scale field exercise showed baseline values starting at level 2 (disagree). This upward shift suggests that repeated exposure to structured scenario-based training produces anticipatory learning effects, thereby increasing baseline preparedness even prior to formal exercise participation.

At item level, five of six evaluated domains demonstrated improvement, with gains ranging from 18.2 to 63.0% following exercise participation (full-scale field exercise, *n* = 17). The mean overall improvement of 34.8 percentage points indicates a robust and consistent enhancement in perceived competencies. In aggregate, post-exercise responses shifted toward stronger agreement, accompanied by a reduction in neutral and negative responses, suggesting consolidation of operational confidence across participants.

These findings were statistically supported by Chi-square analyses, with Fisher's exact test applied where expected cell counts were below threshold. The results confirm significant pre–post differences in response distributions and are consistent with established literature in disaster preparedness research, which demonstrates that repeated, scenario-based, interprofessional training improves both cognitive understanding and operational performance in complex emergency settings. Furthermore, the moderate effect sizes observed in both the second command exercise (*d* = 0.64) and the full-scale field exercise (*d* = 0.56) indicate that these improvements were not only statistically significant but also of practical relevance.

Several limitations of the present exercise evaluation warrant transparent acknowledgment. The sample sizes of 11 and 17 participants, respectively, fall below thresholds conventionally applied in quantitative survey research. However, the study was designed as an exploratory evaluation of a newly developed operational strategy rather than a confirmatory quantitative investigation, and participation was deliberately restricted to stakeholders directly involved in the operational management of infectious mass casualty incidents in the port context, including representatives of the Hamburg Port Authority and its Port Medical Service, the Hamburg Fire and Rescue Service, the Hamburg Authority for Health and Consumer Protection, and representatives of the affected maritime sector. Each participant was nominated by their organization on the basis of professional expertise and operational responsibility, representing a purposive expert sample whose consolidated assessments carry substantive inferential value that cannot be replicated by increasing participant numbers alone. The pool of eligible participants was therefore inherently constrained by the size of the responsible expert community within the study region.

Data collection was conducted during the COVID-19 pandemic, during which public health regulations in Germany imposed legally binding restrictions on group assemblies, rendering the convening of larger cohorts operationally and legally impracticable. The achieved sample sizes consequently represent the maximum permissible under the prevailing regulatory framework, a circumstance entirely beyond the control of the research team. While the pandemic posed substantial challenges to project implementation and evaluation, it simultaneously provided a real-world demonstration of the critical importance of preparedness planning, inter-agency coordination, and response strategies for infectious disease-related mass casualty incidents. Participant numbers and response rates are reported in the Methods section.

The comparatively lower response rate in the final full-scale exercise can be attributed to its hybrid format. Owing to restrictions related to the COVID-19 pandemic, the exercise was conducted partly on-site and partly online, limiting the number of participants who could be physically present. Furthermore, participants involved in the on-site exercise were distributed across different locations within the port terminal, making the immediate post-exercise administration and collection of questionnaires logistically challenging.

Finally, the evaluation instrument was not subjected to formal psychometric validation. Findings should therefore be interpreted as preliminary evidence of feasibility and acceptability, warranting validation in larger and potentially multi-regional exercises in future research.

### Structural and operational framework for maritime MCI-ID response

Due to its spatial configuration, logistical connectivity, and partial separation from densely populated urban areas, a major cruise terminal is particularly well suited for managing large-scale infectious disease incidents in maritime contexts. Its structural characteristics, including isolation potential, multi-berth capacity, and integration into transport and emergency response networks, provide significant operational advantages for scalable disaster response. Within the research consortium, these characteristics were identified as key determinants for its designation as a reference site.

From an infrastructure resilience perspective, these findings underline the necessity of incorporating flexible spatial planning concepts into future port development strategies. In particular, the capacity to rapidly establish isolation zones and deploy temporary medical treatment infrastructure represents a critical determinant of response effectiveness in infectious mass casualty scenarios.

A further limitation of the present study pertains to the deliberate restriction of certain operationally sensitive details in this publication. In accordance with the ethical approval granted by the Commission for the Ethical Evaluation of Research with Potential Dual-Use Risk (reference no. BB 051/19), the specific terminal facility utilized as the primary reference site has not been identified by name, and detailed spatial configuration diagrams of the operational areas have been withheld. These omissions were deemed necessary to mitigate potential dual-use risks associated with the disclosure of critical infrastructure-related information. Whilst this limits the granularity of the methodological description and may constrain direct replicability at the site level, the operational principles, phase-based framework, and strategic recommendations presented herein retain their validity and transferability for analogous port facilities with comparable infrastructural characteristics.

Exercises further demonstrated that Maritime Incident Response Groups (MIRGs), consisting of specialized firefighting units trained for ship-based emergency operations (43), can be deployed to vessels at sea. This enables early triage and clinical assessment on board, facilitating the prioritized evacuation of critically ill patients via helicopter transport. However, this capability remains dependent on robust communication pathways between shipping operators and maritime authorities. Moreover, while such early intervention strategies may improve outcomes in low- to moderate-casualty scenarios, they do not resolve the fundamental structural limitation of maritime settings: the disproportion between patient numbers and available medical resources.

### Coordination structures, patient flow, and public health integration

Upon vessel arrival in port (Phase 2), the operational strategy transitions into Phase 3, which comprises full activation of land-based emergency response structures according to established Hamburg disaster management concepts (15, 16). At this stage, a dual leadership model involving a Leading Emergency Physician and a Port Medical Service physician is proposed. This model reflects an integrated disaster medicine approach, combining operational command expertise with specialized public health and infection control competencies.

The LEP contributes extensive knowledge of local MCI operational structures and clinical disaster response, while the PMS provides expertise in infection prevention, epidemiological risk assessment, and regulatory health authority coordination. Importantly, the PMS contributes to the local detection, assessment, reporting, and escalation of potential infectious disease events and supports coordination with the competent public health authorities. The formal determination of a Public Health Emergency of International Concern (PHEIC), however, lies with the WHO Director-General in accordance with the International Health Regulations. This dual structure enhances situational awareness, strengthens inter-organizational coordination, and improves decision-making efficiency under conditions of uncertainty (3, 34, 39, 40).

The conceptual framework of MCI definitions, such as DIN 13050 (12), warrants critical reflection, as it does not distinguish between trauma-related and infectious medical emergencies. From a disaster medicine perspective, this omission is operationally relevant, as both categories differ substantially in triage logic, resource requirements, and infection control considerations. This limitation becomes particularly evident in infectious MCI scenarios, where diagnostic uncertainty and heterogeneous clinical presentations complicate structured response algorithms.

### Operational implementation: patient flow, communication, and system resilience

The implementation of the operational strategy was evaluated through structured exercises at multiple levels, including command-post simulations and full-scale field exercises. The results demonstrate an initial increase in system resilience among participating stakeholders. Importantly, the iterative exercise design fostered both cognitive and organizational learning, leading to improved inter-agency coordination and enhanced shared situational understanding.

From an operational perspective, sequential patient processing based on triage categories, including mandatory disinfection procedures within the terminal “Arrival” area, was identified as resource-intensive but necessary for infection control. While simultaneous evacuation of lower-acuity patients (“yellow” and “green”) could reduce processing time, it introduces significant infection transmission risks. Therefore, a strictly prioritized evacuation strategy based on descending triage categories remains the recommended approach and was consistently validated in exercise scenarios.

In low-caseload situations, modified operational pathways may be feasible, allowing earlier evacuation of lower-acuity groups once critically ill patients are stabilized on board. Effective implementation of such adaptive strategies requires continuous coordination between on-site operational units and central dispatch systems to avoid transport bottlenecks and ensure optimal resource utilization.

Communication with ship command structures represents a critical operational pillar. Transparent and continuous information exchange is essential to maintain order, ensure compliance, and prevent escalation of psychosocial stress among passengers. The exercises further highlighted the supporting role of police forces in maintaining public order during high-stress maritime emergency operations.

### Public health measures, strategic implications, and lessons learned

Public health measures, particularly contact tracing and case documentation, must be systematically integrated into Phase 4 of the operational strategy. Considering the incubation periods of infectious diseases, delayed symptom onset must be anticipated. Accordingly, structured information provision to passengers is essential to ensure post-discharge surveillance and risk awareness.

The separation of medical treatment areas from public health administrative processes has been identified as a key efficiency factor, reducing operational congestion and alleviating psychosocial burden on both passengers and staff. This spatial and functional separation contributes to improved workflow efficiency in large-scale incident management.

From a strategic perspective, the ARMIHN project demonstrates that iterative, cross-sectoral exercise frameworks significantly enhance inter-organizational resilience and operational readiness. While certain infrastructural details remain restricted due to dual-use considerations, the proposed strategy provides a transferable conceptual framework for infectious MCI management in port environments.

The COVID-19 pandemic has further underscored the need to critically reassess quarantine-based response strategies in maritime settings. Evidence suggests that early evacuation of infected individuals may, under certain conditions, be more effective than prolonged on-board isolation (4, 9). Consequently, continuous reassessment of operational doctrine remains essential.

Finally, scalability and transferability of the concept must be further evaluated in scenarios involving high patient loads and resource saturation. Ongoing documentation, systematic evaluation, and international knowledge exchange will be critical for further refinement. Within ARMIHN, these processes are supported by dedicated work packages, including systematic reviews of pandemic response experiences and the ongoing validation of a novel MCI triage scoring system (44).

## Conclusion

The development of an operational strategy for managing an infectious MCI on board a ship within a port environment represents a highly complex challenge, as it requires balancing the heterogeneous needs of multiple end-users, practitioners, and institutional stakeholders. The successful establishment and implementation of such a strategy therefore depends critically on the active involvement of all relevant parties and necessitates continuous training through repeated, scenario-based exercises.

The operational strategy presented in this study is structured into four sequential phases, reflecting the temporal progression of a vessel from initial notification through to land-based emergency response operations. The proposed concept of an “order of space” enables a structured, float-based operational response framework for managing MCI-ID events within the port environment. Within this structure, triage and patient management are organized into two interconnected levels: the medical treatment with initial triage level and the public health authority level. This dual-layered approach represents a promising framework for improving preparedness for infectious MCI events in port settings, as enhanced preparedness among involved stakeholders directly contributes to increased systemic resilience.

A key strength of this approach lies in its capacity to facilitate continuous intra- and inter-organizational exchange. Repeated training exercises and simulations enable the identification of operational gaps and areas for improvement. On this basis, the strategy is intended as an iterative and adaptive process, allowing continuous refinement and optimisation with the overarching aim of strengthening the resilience of critical port infrastructure systems.

Within the ARMIHN project, cross-organizational exercises have already demonstrated that the implementation of the proposed strategy leads to measurable improvements in the management of infectious MCI events. The next logical step is the dissemination of the concept beyond the national context, including international adaptation and validation, as well as the systematic identification of context-specific limitations and optimisation potential.

Ultimately, the continuous, evaluation-driven refinement of the operational strategy across different port environments contributes to the consolidation of the ARMIHN concept: adaptive resilience management in port settings.

## Data Availability

The raw data supporting the conclusions of this article will be made available by the authors, without undue reservation.
