# Supplementary material for: Managing infectious mass casualty incidents in port environments strategy development and evaluation within the ARMIHN project
Source: Front Public Health. 2026 Jul 8;14:1878189. doi: 10.3389/fpubh.2026.1878189 (PMC13388810; doi:10.3389/fpubh.2026.1878189)
Supplement: Supplementary file 1 [file Data_Sheet_1.pdf]

**Pre-Evaluation Questionnaire:**	**Post-Evaluation Questionnaire:**
1. I have a good understanding of the relevant contact persons and their respective responsibilities and competencies in the event of a mass casualty incident (MCI) / mass casualty incident involving infectious diseases (MCI-ID).<br>2. I have a good understanding of the temporal progression and alerting phases in the event of a mass casualty incident (MCI/ MCI-ID).<br>3. I have a good understanding of the existing interfaces and know how to prevent information loss.<br>4. I have basic skills in implementing an operational strategy in the event of a mass casualty incident (MCI/ MCI-ID).<br>5. I would describe the quality of my handling of a mass casualty incident (MCI/ MCI-ID) situation as good.<br>6. I have sufficient confidence in managing a mass casualty incident (MCI/ MCI-ID).<br>7. I have the ability to systematically assess complex crisis situations.<br>8. I have the ability to professionally evaluate complex crisis situations.<br>9. I believe that my team and/ or I are well prepared for a mass casualty incident (MCI/ MCI-ID) on a ship/ in a port.<br>10. From the exercise, I expect to gain increased operational confidence in managing a mass casualty incident involving infectious diseases.<br>11. From the exercise, I expect to gain a better understanding of the existing information pathways.<br>12. From the exercise, I expect to gain a better insight into staff work.<br>13. Age in years (20-30; 30-40;40-50;50-60;60+)<br>14. Experience in the current profession in years (0-5; 5-10; 10+)<br>15. My prior experience in the field of mass casualty incidents (MCI)/ mass casualty incidents involving infectious diseases (MCI-ID) is: (extensive – more than 3 deployments; moderate – 1–3 deployments; limited – no deployments).<br>16. My prior experience in training for mass casualty incidents (MCI)/ mass casualty incidents involving infectious diseases (MCI-ID) is: (extensive – more than 3 training sessions; moderate – 1–3 training sessions; limited – no training).<br>17. Highest educational qualification (lower secondary school certificate, intermediate secondary school certificate, (specialised) higher education entrance qualification / university degree).	1. I have a good understanding of the relevant contact persons and their respective responsibilities and competencies in the event of a mass casualty incident (MCI) / mass casualty incident involving infectious diseases (MCI-ID).<br>2. I have a good understanding of the temporal progression and alerting phases in the event of a mass casualty incident (MCI/ MCI-ID).<br>3. I have a good understanding of the existing interfaces and know how to prevent information loss.<br>4. I have basic skills in implementing an operational strategy in the event of a mass casualty incident (MCI/ MCI-ID).<br>5. I would describe the quality of my handling of a mass casualty incident (MCI/ MCI-ID) situation as good.<br>6. I have sufficient confidence in managing a mass casualty incident (MCI/ MCI-ID).<br>7. I have the ability to systematically assess complex crisis situations.<br>8. I have the ability to professionally evaluate complex crisis situations.<br>9. I believe that my team and/ or I are well prepared for a mass casualty incident (MCI/ MCI-ID) on a ship/ in a port.<br>10. The exercise was well structured.<br>11. The exercise was well organized.<br>12. The scenario was realistic.<br>13. The pre-exercise briefing was useful and prepared me for the exercise.<br>14. The exercise enabled us to test our emergency plans and systems.<br>15. The exercise improved my understanding of my role and function during an infectious disease emergency.<br>16. The exercise helped me identify some of my strengths as well as gaps in my understanding of emergency systems, plans, and procedures.

*Items 4–9 were included in the quantitative analysis
